# PRACTICE-HF: Implementation of an updated clinical protocol for acute heart failure

**DOI:** 10.1007/s12471-025-02004-8

**Published:** 2026-01-05

**Authors:** Lukas Peeters, Mick Hoen, Delian Hofman, Bjorn Hompes, Bart Langenveld, Danae Smeets, Timo Lenderink, Hans Peter Brunner-La Rocca, Sandra Sanders-van Wijk

**Affiliations:** Department of cardiology, Zuyderland Medical Centre, Heerlen, The Netherlands

**Keywords:** Heart failure, Clinical implementation, Guideline-directed medical therapy, ARNI, SGLT2i, Iron

## Abstract

**Background:**

Despite recent advances in the treatment of acute heart failure (AHF), implementation of new evidence into clinical practice remains challenging.

**Methods:**

We conducted a single-center descriptive exploratory study within an ongoing prospective AHF registry. Adult patients admitted with AHF, without requiring intensive care, were included consecutively. An updated local AHF protocol was developed and implemented by group-education sessions, pocket cards, and posters. Patients before (control group) and after (intervention group) implementation of the new protocol were compared in terms of compliance to the protocol and 90-day outcomes—blanking the implementation period. Subgroups entailed HF with (mildly) reduced and preserved ejection fraction.

**Results:**

Patients were elderly, with almost half being de novo HF patients. Groups were comparable except for higher NT-proBNP in the implementation group and a higher cancer prevalence in the control group. The intervention group showed an increase in in-hospital use of acetazolamide (59.8 vs 0%, *p* < 0.001), in iron deficiency testing and correct iv. iron administration (42.9% vs 78.6% *p* ≤ 0.001). Pre-discharge installation of SGLT2 inhibitors showed a positive trend (44.2 vs 20% in HF(m)rEF patients and 29.4 vs 4% in HFpEF, both *p* = 0.01) HF-event-free survival at 90 days numerically favored the intervention group (29.9 vs 44.3%, *p* = 0.054), whereas length of hospital stay increased by 1 day (*p* = 0.011).

**Conclusion:**

Implementing a local updated AHF protocol improved adoption of several evidence based AHF interventions. This may translate into improved patient outcomes, against a minor increase in hospital duration.

## What’s new?

- An updated acute heart failure protocol based on the most relevant literature available at the time was developed and implemented into clinical practice.
- Implementing an updated acute heart failure protocol into clinical practice led to more frequent administration of iv. acetazolamide, and correct iv. iron administration. Trends towards improved SGLT2‑i prescription, but also increase in hospitalization duration of +1 day were observed.

## Introduction

Acutely decompensated Heart Failure (AHF) is notorious for its high re-hospitalization rate and adverse prognosis [1] Adequate treatment to reduce morbidity is thus essential. Loop diuretic treatment for decongestion—and hemodynamic stabilization—remained standard of care for a long period of time, with scarce evidence supporting other interventions in AHF. Additionally, initiation of evidence-based drugs for chronic HF is an important treatment target during hospitalization for AHF [2] Advances were made in the treatment of AHF, resulting in a position statement on diuretic therapy and an update of the European Society of Cardiology (ESC) guideline [3, 4] More advances were published after 2021, [2, 5–8]—leading to another update of the ESC HF guideline in 2023 [9] These guidelines have a supportive role in clinical practice. Actual implementation of new evidence is slow, and adoption is often incomplete. For example, the CHECK-HF study revealed that the average percentage of optimally dosed heart failure drugs was low (43.6%, 18.9% and 52% for ACE-inhibitors (ACE-i)/Angiotensin receptor blockers (ARB), Beta-blockers, and mineralocorticoid receptor antagonists (MRA), respectively) despite ample time for adoption [10] Implementation of new evidence during the critical AHF phase may be especially challenging due to heterogeneous patient presentation and diversity of hospital settings, whilst this vulnerable phase should be considered an opportunity to optimize HF-therapy. There is scarce data on implementation strategies for AHF treatment since the new guidelines. Therefore, we evaluated the implementation of an updated AHF protocol based upon the most recent scientific evidence on AHF treatment available at the time.

## Methods

### Study design and AHF protocol

We conducted a single-center descriptive exploratory study, within an ongoing, prospective AHF registry, which started in Oct 2021. From Feb-May 2022, we developed a local AHF protocol in the form of posters and pocket-cards, following the updated ESC guidelines [3] the 2019 HFA position statement [3] and other published randomized trials at the time [2, 5, 7, 8] It was updated further in late August 2022 to include addition of acetazolamide for decongestion, and SGLT2‑I prescription for HFpEF, following results from the ADVOR and DELIVER trial, respectively [11, 12].

**Fig. 1:**
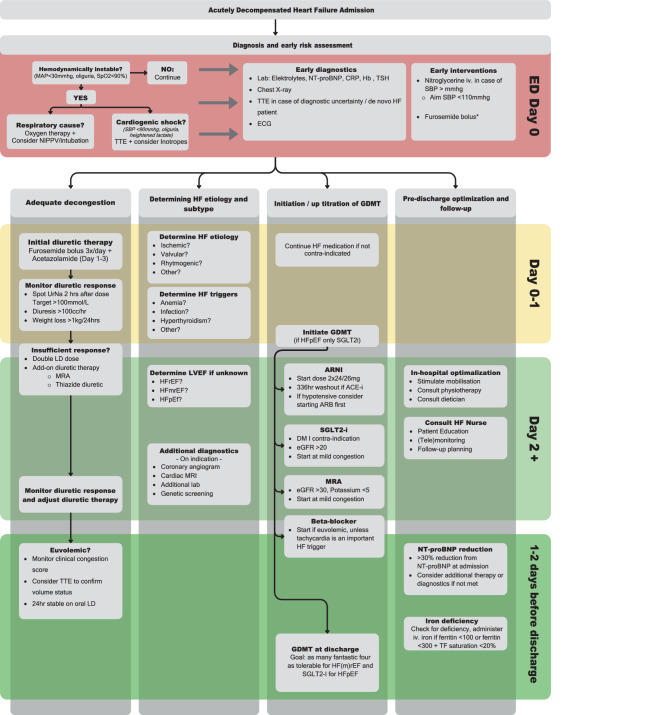
Summarized updated clinical AHF protocol. (*NIPPV* Noninvasive positive pressure ventilation, *TTE* Trans-thoracic echocardiogram, *ECG* Electrocardiogram, *HF* Heart failure, *NT-proBNP* N-terminal pro‑B type natriuretic peptide, *CRP* C-reactive protein, *Hb* Hemoglobin, *TSH* Thyroid-stimulating hormone, *Iv* Intravenous, *SBP* Systolic blood pressure, *ED* Emergency department, *GDMT* Guideline-directed medical therapy, *MAP* Mean arterial pressure, *MRA* Mineralocorticoid receptor antagonists, *SGLT2‑i* Sodium glucose transporter−2 inhibitor, *ARNI* Angiotensin receptor/neprilysin inhibitor, *ACE‑i* Angiontensin-converting enzyme inhibitory, *LVEF* Left ventricular ejection fraction, *HF* *(m)rEF* Heart failure with (medium) reduced ejection fraction, *HFpEF* Heart failure with preserved ejection fraction, *VCI* Vena cava inferior, *LD* Loop diuretic. *First furosemide bolus administration according to a starting dose dosage table)

An overview of our AHF protocol is illustrated in Fig. 1. The protocol focuses on five domains: 1) Heart failure (HF) diagnosis and early risk assessment; 2) Adequate and timely decongestion; 3) Determining HF etiology, trigger and HF subtype (i.e., Heart failure with reduced ejection fraction (HFrEF), Heart failure with mildly reduced ejection fraction (HFmrEF) or heart failure with preserved ejection fraction (HFpEF); 4) Early initiation/uptitration of guideline-directed medical therapy (GDMT) for HF; 5) Pre-discharge and follow-up optimization. For example, the upfront use of acetazolamide for early decongestion and iron testing before discharge. The entire protocol is presented as Fig. 1. Our AHF protocol was implemented in Sept. 2022—as described below. The control group consisted of patients admitted from October 2021 until January 2022. Patients in the implementation group were included from Nov 2022 until Feb 2023. In-between, a blanking period was installed.

### Study population

Patients eligible for inclusion were adults hospitalized with the diagnosis of AHF according to the 2021 ESC HF guidelines. Patients could be included regardless of HF etiology, left-ventricular ejection fraction (LVEF) or previous diagnosis of HF. Patients admitted to the intensive care were excluded.

#### Implementation—physician instruction and training

To implement our AHF protocol and attain adherence, physicians, residents, and specialized nurses working in the cardiology clinic were instructed and educated in employing the protocol by means of repeated group education sessions and individual sessions with new employees thereafter. Pocket versions of the protocol were provided, and A0-size posters of the protocol were located at all working stations to improve and retain protocol adherence.

### Outcomes of interest

The primary outcome of interest was improved uptake in domain 4, since the evidence for BB/MRA/SGLT2-i/ARNI in HF(m)rEF and SLGT2‑i in HFpEF is most robust. The outcome was defined as the installation of 3 or more HF-drug classes in HF(m)rEF and installation of SGLT2‑i in all types of HF. Secondary outcomes of interest were outcomes in the other domains of our protocol. More specifically, i) installation of acetazolamide (domain 2), ii) absolute iv. iron administration pre-discharge (domain 5). Exploratory, we evaluated i) hospital duration, ii) in-hospital mortality, iii) HF-event-free survival at 90-day follow-up, in which HF-events consisted of HF-rehospitalizations combined with outpatient iv. diuretic administration and outpatient loop diuretic doubling. These events were adjudicated by the research team. iv) number of outpatient visits/contacts within 90-days follow up and v) achievement of a 30% NT-proBNP reduction from admission to discharge.

### Ethics and data collection

This trial was designed and conducted in accordance with the Declaration of Helsinki. The study was approved by the local medical ethics committee. All patients provided written informed consent. Data was collected from the electronic patient file.

### Statistical analysis

We present results as mean (± standard deviation), median (25th–75th percentile) or frequency (%). Between-group comparisons were performed using independent samples T‑tests, Chi-square, or Mann-Whitney U tests as appropriate. A 2-sided *p*-value of 0.002 (corrected for multiple testing using the Bonferroni correction) was considered statistically significant. All statistical analyses were performed using IBM SPSS Statistics Version 29.0.

## Results

### Baseline characteristics

Our population consisted of 166 patients, being a representative comorbid elderly population, of whom roughly half presented with HF de novo, and > 50% presented with NYHA class III–IV. Baseline characteristics are presented in Tab. 1.

**Table 1:** Study population baseline characteristics

Characteristic	Control group (*n* = 79)	Implementation group (*n* = 87)	Valid *N* (%)	*P*-value
Age (yrs), mean (Std. dev.)	77 (± 11)	79 (± 9)	166	0.225
Female	32 (40.5%)	43 (49.4%)	166	0.249
*NYHA*			127 (76.5%)	0.457
Class I	4 (5.1%)	3 (3.4%)		
Class II	13 (16.5%)	9 (10.3%)		
Class III	28 (35.4%)	29 (33.3%)		
Class IV	20 (25.3%)	21 (24.1%)		
Systolic BP—mean (Std. dev.)	135 (± 26)	139 (± 25)	166	0.274
Diastolic BP—mean (Std. Dev.)	78 (± 18)	78 (± 18)	166	0.972
Heart rate—mean (Std. dev.), beats/min	92 (± 23)	91 (± 24)	166	0.972
eGFR, median (25th–75th percentile)—ml/min/1.73 m2	51 (34–69)	48 (30–75)	166	0.522
Serum creatinine, median (25th–75th percentile)—umol/l	107 (82–157)	107 (75–156)	166	0.874
NTproBNP, median (25th–75th percentile) pG/mL	5039 (2749–13,119)	8518 (2995–19,634)	161 (97%)	0.049
Heart failure, *n* (%)	39 (49.4%)	43 (49.4%)	166	0.994
*HF-Type, (% of previously known HF patients)*				
HFpEF	11 (28.2%)	16 (37.2%)		0.621
HFmrEF	8 (20.5%)	10 (23.3%)		
HFrEF	18 (46.1%)	16 (37.2%)		
Unknown	2 (5.1%)	1 (2.3%)		
Significant CAD	31 (39.2%)	30 (34.5%)	166	0.783
ACS	16 (20.3%)	21 (24.1)%	166	0.548
Diabetes	32 (40.5%)	31 (35.6%)	166	0.518
Atrial fibrillation	48 (60.8%)	48 (55.2%)	166	0.467
Kidney disease	25 (31.6%)	33 (37.9%)	165 (99.4%)	0.366
Hypertension	47 (59.5%)	57 (65.5%)	166	0.423
Hypercholesterolaemia	28 (35.4%)	24 (27.6%)	166	0.276
Stroke	14 (17.7%)	10 (11.5%)	166	0.255
Peripheral artery disease	10 (12.7%)	10 (11.5%)	166	0.818
Pulmonary hypertension	6 (7.6%)	3 (3.4%)	166	0.239
COPD	18 (22.8%)	14 (16.1%)	166	0.275
Obstructive sleep apnea	10 (12.7%)	12 (13.8%)	166	0.829
Anaemia	22 (27.8%)	22 (25.3%)	166	0.709
Iron deficiency	9 (11.4%)	10 (11.5%)	166	0.984
Cancer	26 (32.9%)	16 (18.4%)	166	0.032
Loop diuretic	48 (60.8%)	51 (58.8%)	161 (97%)	0.978
Thiazide diuretic	3 (3.8%)	13 (14.9%)	164 (98.8)	0.013
Beta-blocker	42 (53.2%)	47 (54%)	165 (99.4)	0.848
ACE‑I	22 (27.8%)	21 (24.1%)	165 (99.4)	0.616
ARB	15 (19%)	18 (20.7%)	165 (99.4)	0.755
MRA	20 (25.3%)	20 (23.0%)	165 (99.4)	0.758
SGLT2i	1 (1.3%)	11 (12.6%)	165 (99.4)	0.004
ARNI	0 (0%)	6 (6.9%)	165 (99.4)	0.017

*Std. dev* Standard deviation, *NYHA* New York Heart association, *BP* Blood pressure, *eGFR* Estimated glomerular filtration rate, *NT-proBNP* N-terminal pro‑B type natriuretic peptide, *CAD* Coronary-artery disease, *ACS* Acute coronary syndrome, *COPD* Chronic obstructive pulmonary disease, *MRA* Mineralocorticoid receptor antagonists, *SGLT2‑i* Sodium glucose transporter−2 inhibitor, *ARNI* Angiotensin receptor/neprilysin inhibitor, *ACE‑i* Angiotensin-converting enzyme inhibitor, *ARB* Angiotensin receptor blocker

### Protocol adherence

#### Domain 2: *Adequate and timely decongestion*

The management of acute decongestion is shown in Tab. 2. Uptake of upfront acetazolamide was > 50% in the intervention group and was not yet used in the control group. Duration and cumulative dosage of iv. Loop diuretics did not differ between groups. Hydrochlorothiazide installation was limited and similar between groups.

**Table 2:** Outcomes on adequate and timely decongestion in the control and implementation group

Outcome	Control group (*n* = 79)	Implementation group (*n* = 87)	*P*-value
Acetazolamide use	0 (0%)	52 (59.8%)	< 0.001
Hydrochlorothiazide use	3 (3.8%)	2 (2.3%)	0.573
Cumulative iv. loop diuretic use, (mg.) Mean (95% CI)	814 (523–1105)	1017 (710–1325)	0.187
Iv. loop diuretic duration, median (IQR) (Days)	3 (1–6)	4 (2–7)	0.408

*Iv* Intravenous, *IQR* interquartile range, *Mg* Milligrams, *CI* Confidence interval

#### Domain 4: *Initiation/uptitration of GDMT*

**Fig. 2:**
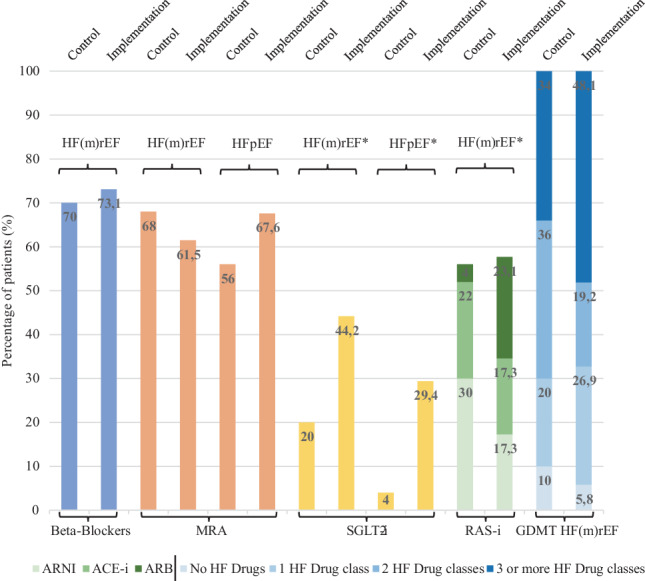
Guideline-directed medical therapy, prescription rates and quantity of prescribed GDMT drugs at discharge. (*MRA* Mineralocorticoid receptor antagonists, *SGLT2‑i* Sodium glucose transporter−2 inhibitor, *ARNI* Angiotensin receptor/neprilysin inhibitor, *ACE‑i* Angiotensin-converting enzyme inhibitor, *ARB* Angiotensin receptor blocker, *RAS‑i* Renin angiotensin system inhibition, *GDMT* Guideline-directed medical therapy, *HF* *(m)rEF* Heart failure with (medium) reduced ejection fraction, *HFpEF* Heart failure with preserved ejection fraction. *P-value ≤ 0.05 was found, **P-value ≤ 0.002 was found, thus signifying a statistical significant difference)

In both HF(m)rEF and HFpEF patients, SGLT2i uptake showed a trend in improvement (Fig. 2). In HF(m)rEF patients, installation of 3 or more GDMT HF drug classes increased numerically to 48.1% in the intervention group compared to 34% in the control group, possibly driven by an increase in SGLT2-inhibitor prescription rates. The overall prescription rate of ACEi/ARB and angiotensin receptor/neprilysin inhibitor (ARNI) was similar between groups whilst the distribution of individual drug classes differed, showing less ARNI but more ARB prescription in the intervention group.

#### Domain 5: *Pre-discharge and follow-up optimization*

**Fig. 3:**
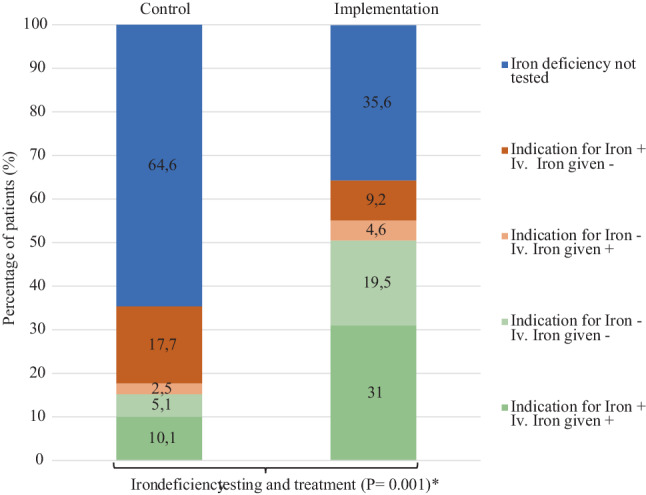
Iron deficiency testing and treatment pre-discharge between research groups. *In this figure, the combination of green and red elements of the stacked column represent the patients where iron deficiency testing was performed during admission. It is then further subdivided whether patients had an indication for intravenous iron administration and whether or not they received iv. iron during admission. Indication for iv. iron administration was defined according to guidelines (i.e. serum ferritin < 100 ng/ML or 100–299 ng/mL with TSAT < 20%)

Iron deficiency testing almost doubled, up to 60% in the intervention group. Intravenous (iv.) iron administration also doubled in the implementation group (Fig. 3). Rates of correct execution ofiv. iron administration (i.e, patients that received iron when indicated or did not receive iron when not indicated) in patients tested for iron deficiency improved (42.9% vs 78.6%, *p* ≤ 0.001). A reduction of > 30% in NTproBNP was achieved similarly in both groups (63.4% vs 64.4%, *p* = 0.803). In-hospital involvement of an HF nurse showed a trend in improvement but remained relatively low (0% vs 10.3%, *P* = 0.003).

### Clinical outcomes

The combined endpoint of HF-event-free survival at 90 days showed a trend favoring the intervention group (Tab. 3). Duration of hospitalization appeared to increase by median +1 day in the implementation group. In-hospital and 90-day mortality rates were similar.

**Table 3:** Clinical endpoints between both research groups.

Outcome	Control group *n* = 79	Implementation group *n* = 87	Double sided *P*-value
Duration of hospitalization, median (25th–75th percentile), days	7 (4–10)	8 (5–15)	0.011
In-hospital mortality-rate, *n* (% of mortality)	4 (23.5%)	1 (5.9%)	0.126
HF-events + all-cause mortality 90-days post discharge*, *n* (%)	35 (44.3%)	26 (29.9%)	0.054
All-cause mortality 90-days post discharge, *n* (%)	17 (21.5%)	17 (19.5%)	0.793
All-cause rehospitalizations 90-days post discharge, *n* (%)	24 (30.3%)	22 (25.9%)	0.108
Outpatient visits + phone consultations 90-days post-discharge, Median, (25th–75th percentile)	1 (0–3)	1 (0–3)	0.141

*HF-events consists of HF-rehospitalization 90-days post discharge + outpatient iv. diuretic administration and outpatient oral diuretic doubling

## Discussion

Our results show that a simple implementation strategy of new AHF evidence, improved iron deficiency testing and treatment in patients hospitalized for AHF. Furthermore, protocol implementation led to more aggressive decongestive therapy with a marked increase in upfront use of acetazolamide. Diuretic iv. duration, cumulative diuretic dose and delta NT-proBNP during hospitalization did not change. Overall, HF-drug prescription did not seem to change, however SGLT‑2 prescription at discharge showed a trend towards improvement [11].

### Implementation of an updated AHF protocol in clinical practice

#### GDMT

Beta blockers, MRA’s, ACEi/ARB/ARNI and SGLT2-inhibitors are the cornerstone treatment for patients with HFrEF [3]. STRONG-HF revealed that rapid up-titration of these drugs during and shortly after hospitalization results in improvements in quality of life, heart failure symptoms, and 180-day HF hospitalization-free survival [2]. Before protocol implementation, prescription rates at discharge for these drugs were relatively high, except for ARNI and SGLT2i. A trend towards increased SGLT2-inhibitor prescription was seen after protocol implementation. Surprisingly, distribution of RAS-inhibition differed, with a trend towards lower ARNI prescription rates after protocol implementation. We hypothesize that this is largely due to a reimbursement limitation on ARNI, installed in June 2022 by the ZIN (Zorg Instituut Nederland) [13]—which was effective throughout the entire post-implementation period of our study. It restricted reimbursement of clinical ARNI prescription in ACE-i/ARB naïve patients. This hypothesis is supported by a higher prescription rate of ARB during protocol implementation. The prevalence of triple therapy at discharge was 48.1% in the implementation group (in HF(m)rEF patients) compared to 34.5% in the HELP-HF registry [14]. This slight improvement is consistent with the recent results of the IMPLEMENT-HF trial, which showed that GDMT improved after implementation initiatives and efforts for quality improvement [15].

#### Adequate and timely decongestion

Residual congestion at discharge is associated with higher re-hospitalization rates and death [16] Our protocol thus advised the addition of acetazolamide early during admission, following early available results of the ADVOR trial [11]. The marked increase in acetazolamide use suggests that implementation of our protocol improves translation of scientific evidence into real-world use. Our protocol advised loop diuretic doubling or diuretic combination therapy in case of inadequate diuretic response, including timely evaluation of diuretic response using combined measures, complying with the ESC HF working group position statement [4] Whether our successful implementation of diuretic combination therapy resulted in more adequate decongestion cannot be determined, due to missing values caused by variable documentation of decongestion parameters.

#### Pre-discharge assessment and optimization

Our protocol advised physicians to optimize patients 1–2 days before discharge. Iron deficiency testing and subsequent iv. administration when indicated, adequate follow-up planning and reduction of NTproBNP of > 30% were emphasized as key focus points before discharge. The prevalence of iron deficiency is high in patients with AHF, with prevalence at discharge reaching up to 55% [17]. The recent AFFIRM-AHF trial revealed that stabilized AHF patients with iron deficiency and reduced ejection fraction benefitted from iv. iron administration, leading to reduced HF rehospitalizations [5]. The cut-off values for iv. iron administration in our protocol was upheld according to the most recent guidelines published. Iron deficiency testing and iv. iron administration improved after protocol implementation, and fewer patients received iv. iron incorrectly—i.e. not having an indication. This is most likely due to the increased iron deficiency testing during admission.

Following the ESC guidelines [3] and a meta-analysis conducted by Van Spall et al. [18], our protocol advised consulting a specialized HF nurse during admission. The aim was to improve patient-education, (tele)monitoring and routine follow-up after discharge. Although HF-nurse consultations during hospitalization improved, it was still limited. This occurred most likely due to understaffing.

#### Clinical outcomes

A positive trend in clinical outcomes was observed, but these results are exploratory only. This trend could be caused by improvements in the installation of evidence-based HF therapies. Which part of our protocol was responsible for this trend cannot be concluded from this study. Compared to REPORT-HF, our study shows similar rehospitalization yet higher mortality rates [19] This could be explained by our population being approximately ten years older compared to REPORT-HF. We did see a trend in an increase of hospitalization duration of +1 day (median), whereas the duration of iv. diuretic treatment was similar. This longer admission time was surprising, since the PUSH-HF and ADVOR trial revealed a reduction in admission time [11, 20]. The exact cause of this increase remains undetermined, our main hypothesis being that by optimizing GDMT and additional interventions, hospitalization duration lengthened. Although statistically non-significant, a possible increase in hospitalization time should be taken into consideration while implementing our protocol. This could add to the workload of HF healthcare. Care-consumption measured by the number of outpatient-contacts during 90-days follow-up was similar between groups. Finally, our results reinforce the need for further implementation studies and real-world data on advances in AHF care.

#### Practical recommendations

Based upon our and previous results, some practical recommendations are suggested to optimize clinical implementation of new evidence concerning HF: i) Develop, distribute and implement a local AHF-protocol, our protocol—Fig. 1 could be used as a template; ii) regularly educate physicians, concerning the latest scientific evidence and guidelines, iii) provide easy/clear access to guidelines/latest scientific evidence (e.g. posters in working spaces, pocket cards).

## Limitations

Due to the exploratory nature of our study, interpretation of the results of our statistical analyses should be made with caution. Insufficient data could be collected from to electronic patient files to analyze outcomes on domains 1 and 3 of our protocol. Due to our study being a single-center study conducted in the Netherlands, our AHF protocol could lose applicability in different countries/health care systems and should be adjusted accordingly. Our study had a relatively small population, resulting in low power for our analysis on clinical outcomes. This calls for larger-scale research on the implementation of AHF treatment strategies. Finally, we updated our local AHF protocol as appropriate using the available literature at the time (2022). A standardized method to integrate new evidence into our protocol was not developed.

## Conclusion

Implementing a local updated AHF protocol improved adoption of several evidence-based AHF interventions. This may translate into improved patient outcomes, against a minor increase in hospital duration.

## References

[1] Naderi N, Chenaghlou M, Mirtajaddini M, et al. Predictors of readmission in hospitalized heart failure patients. J Cardiovasc Thorac Res. 2022;14(1):11–7. 35620751 10.34172/jcvtr.2022.08 PMC9106947

[2] Mebazaa A, Davison B, Chioncel O, et al. Safety, tolerability and efficacy of up-titration of guideline-directed medical therapies for acute heart failure (STRONG-HF): a multinational, open-label, randomised, trial. Lancet. 2022;400(10367):1938–52. 36356631 10.1016/S0140-6736(22)02076-1

[3] McDonagh TA, Metra M, Adamo M, et al. 2021 ESC Guidelines for the diagnosis and treatment of acute and chronic heart failure. Eur Heart J. 2021;42(36):3599–726. 34447992 10.1093/eurheartj/ehab368

[4] Seferovic PM, Ponikowski P, Anker SD, et al. Clinical practice update on heart failure 2019: pharmacotherapy, procedures, devices and patient management. An expert consensus meeting report of the Heart Failure Association of the European Society of Cardiology. Eur J Heart Fail. 2019;21(10):1169–86. 31129923 10.1002/ejhf.1531

[5] Ponikowski P, Kirwan B, Anker SD, et al. Ferric carboxymaltose for iron deficiency at discharge after acute heart failure: a multicentre, double-blind, randomised, controlled trial. Lancet. 2020;396(10266):1895–904. 33197395 10.1016/S0140-6736(20)32339-4

[6] Clephas PRD, Malgie J, Schaap J, et al. Guideline implementation, drug sequencing, and quality of care in heart failure: design and rationale of TITRATE-HF. Esc Heart Fail. 2024;11(1):550–9. 38064176 10.1002/ehf2.14604 PMC10804201

[7] Biegus J, Voors AA, Collins SP, et al. Impact of empagliflozin on decongestion in acute heart failure: the EMPULSE trial. Eur Heart J. 2023;44(1):41–50. 36254693 10.1093/eurheartj/ehac530 PMC9805406

[8] Berg DD, Samsky MD, Velazquez EJ, et al. Efficacy and safety of sacubitril/valsartan in high-risk patients in the PIONEER-HF trial. Circ Heart Fail. 2021;14(2):e7034. 33530704 10.1161/CIRCHEARTFAILURE.120.007034 PMC7908815

[9] McDonagh TA, Metra M, Adamo M, et al. Focused update of the 2021 ESC Guidelines for the diagnosis and treatment of acute and chronic heart failure. Eur Heart J. 2023;44(37):3627–39. 37622666 10.1093/eurheartj/ehad195

[10] Brunner-La Rocca H, Linssen GC, Smeele FJ, et al. Contemporary drug treatment of chronic heart failure with reduced ejection fraction: the CHECK-HF Registry. JACC Heart Fail. 2019;7(1:13–21. 30606482 10.1016/j.jchf.2018.10.010

[11] Mullens W, Dauw J, Martens P, et al. Acetazolamide in acute decompensated heart failure with volume overload. N Engl J Med. 2022;387(13):1185–95. 36027559 10.1056/NEJMoa2203094

[12] Solomon SD, McMurray JJV, Claggett B, et al. Dapagliflozin in heart failure with mildly reduced or preserved ejection fraction. N Engl J Med. 2022;387(12):1089–98. 36027570 10.1056/NEJMoa2206286

[13] Zorginstituut Nederland. Verslag van de vergadering van de Wetenschappelijke Adviesraad (WAR) over sacubitril/valsartan (Entresto®). Zorginstituut Nederland. 2022;14/11.

[14] Tomasoni D, Pagnesi M, Colombo G, et al. Guideline-directed medical therapy in severe heart failure with reduced ejection fraction: an analysis from the HELP-HF registry. Eur J Heart Fail. 2024;26(2):327–37. 37933210 10.1002/ejhf.3081

[15] Sauer AJ, Beon C, Cherkur S, et al. Multiregional implementation initiative’s impact on guideline-based performance measures for patients hospitalized with heart failure: IMPLEMENT-HF. Circ Heart Fail.0(0):e12547–0. 10.1161/CIRCHEARTFAILURE.124.012547 PMC12084012 40115978

[16] Rubio-Gracia J, Demissei BG, Ter Maaten JM, et al. Prevalence, predictors and clinical outcome of residual congestion in acute decompensated heart failure. Int J Cardiol. 2018;258:185–91. 29544928 10.1016/j.ijcard.2018.01.067

[17] van Dalen DH, Kragten JA, Emans ME, et al. Acute heart failure and iron deficiency: a prospective, multicentre, observational study. Esc Heart Fail. 2022;9(1):398–407. 34862747 10.1002/ehf2.13737 PMC8788059

[18] Van Spall HGC, Rahman T, Mytton O, et al. Comparative effectiveness of transitional care services in patients discharged from the hospital with heart failure: a systematic review and network meta-analysis. Eur J Heart Fail. 2017;19(11):1427–43. 28233442 10.1002/ejhf.765

[19] Tromp J, Bamadhaj S, Cleland JGF, et al. Post-discharge prognosis of patients admitted to hospital for heart failure by world region, and national level of income and income disparity (REPORT-HF): a cohort study. Lancet Glob Health. 2020;8(3):e411–20. 32087174 10.1016/S2214-109X(20)30004-8

[20] Ter Maaten JM, Beldhuis IE, Van Der Meer P, et al. Natriuresis-guided diuretic therapy in acute heart failure: a pragmatic randomized trial. Nat Med. 2023;29(10):2625. 37640861 10.1038/s41591-023-02532-z PMC10579092

